# Facile Synthesis of Phosphatidyl Saccharides for Preparation of Anionic Nanoliposomes with Enhanced Stability

**DOI:** 10.1371/journal.pone.0073891

**Published:** 2013-09-12

**Authors:** Shuang Song, Ling-Zhi Cheong, Mia Falkeborg, Lei Liu, Mingdong Dong, Henrik Max Jensen, Kresten Bertelsen, Michael Thorsen, Tianwei Tan, Xuebing Xu, Zheng Guo

**Affiliations:** 1 Department of Engineering, Aarhus University, Aarhus, Denmark; 2 College of Life Science and Technology, Beijing University of Chemical Technology (BUCT), Beijing, China; 3 Interdisciplinary Nanoscience Center (iNANO), Aarhus University, Aarhus, Denmark; 4 Advanced Analysis, DuPont Nutrition & Health, Brabrand, Denmark; National University of Ireland, Galway, Ireland

## Abstract

Physical stability during storage and against processing such as dehyration/rehydration are the cornerstone in designing delivery vehicles. In this work, mono-, di- and tri-saccharides were enzymatically conjugated to phosphatidyl group through a facile approach namely phospholipase D (PLD) mediated transphosphatidylation in a biphasic reaction system. The purified products were structurally identified and the connectivities of carbohydrate to phosphatidyl moiety precisely mapped by ^1^H, ^31^P, ^13^C NMR pulse sequences and LC-ESI-FTMS. The synthetic phosphatidyl saccharides were employed as the sole biomimetic component for preparation of nanoliposomes. It was found that the critical micelle concentration (CMC) of phosphatidyl saccharides increases as more bulky sugar moiety (mono- to tri-) is introduced. Phosphatidyl di-saccharide had the largest membrane curvature. In comparison to the zwitterionic phosphatidylcholine liposome, all phosphatidyl saccharides liposomes are anionic and demonstrated significantly enhanced stability during storage. According to the confocal laser scan microscopy (CLSM) and atom force microscopy (AFM) analyses, the nanoliposomes formed by the synthetic phosphatidyl saccharides also show excellent stability against dehydration/rehydration process in which most of the liposomal structures remained intact. The abundance hydroxyl groups in the saccharide moieties might provide sufficient H-bondings for stabilization. This work demonstrated the synthesized phosphatidyl saccharides are capable of functioning as enzymatically liable materials which can form stable nanoliposomes without addition of stabilizing excipients.

## Introduction

Phospholipids-based liposomal vesicles have evolved as a promising tool for delivery of drug, genes and nutrients due to intensive investigations for decades [1-4]. Stability of liposomes has been a key issue which limit its application as most liposomes with phosphatidylcholine (PC) as primary components have low stability [4-6]. To circumvent this problem, different strategies have been attempted including development of polymeric liposomes [5-7], functionalization of liposome surface [8-10], synthesis of novel liposome constituting components [11,12], and addition of protectants [13-15]. Addition of protectants such as trehalose and proline has been shown to significantly enhance the stability of liposomes during storage and against dehydration/rehydration process [13-17]. These protectants were hypothesized to act as water replacement molecules which form hydrogen bonding with the phospholipids to maintain head group spacing and thus prevent fusion of liposomes [18,19].

Based on this concept, we hypothesize that conjugation of phosphatidyl and sugar moiety may integrate the function of PC as building block for liposome and the function of protectants as physical stability enhancer into phosphatidyl saccharides. In this work, we had developed a facile approach to conjugate mono-, di- and tri-saccharides to phosphatidyl group through phospholipase D mediated transphosphatidylation in a biphasic reaction system and characterized phosphatidyl saccharide-based liposomes systemically with PC-liposomes as a comparison control. The synthetic phosphatidyl saccharides were found to be capable of forming nanoliposomes with enhanced physical stability against fusion and aggregation following storage, dehydration and rehydration procedures.

This work presented a good demonstration that there is a huge potential of using natural compounds as building blocks to develop low toxic, biodegradable and biocompatible excipients for a variety of applications.

## Materials and Methods

### Materials

Phospholipase D (Streptomyces sp.) was a gift from Nagase ChemteX Corporation, Japan. The activity of the PLD is 600,000 U/g (1 U is defined as the activity which liberates 1 µmol choline from L-α-phosphatidylcholine per minute). Phosphatidylcholine (PC), (Epikuron 200), was a gift from Cargill Inc. (Minneapolis, MN, USA). This PC originates from soybean and consists of at least 95% PC of which 3% is the lyso- form. All solvents were purchased from Sigma-Aldrich (St. Louis, MO, USA) and of HPLC grade. Glucose, sucrose, raffinose and other chemicals were of analytical grade from Sigma-Aldrich Co. (St. Louis, MO, USA).

### Enzymatic Synthesis of Phosphatidyl Saccharides

PLD-catalyzed transphosphatidylation reaction between PC and saccharides was carried out using a biphasic reaction system in which 390 mg of PC was dissolved in 20 ml ethyl acetate and 60 U PLD was dissolved in 2 ml sodium acetate buffer (pH 5.6 adjusted by acetic acid). Firstly, PLD buffer solution was added to reactor with 1.8 g glucose/ 3.42 g sucrose/5.04 g raffinose and stirred for 10 min. Then, PC solvent solution was added in the reactor to initiate the reaction. The reaction was carried out at 50 °C and stirred with magnetic stirrer (600 rpm) for 2 h. At the end of the reaction, 20 ml chloroform was added to the reaction mixture to stop the reaction and extract out the lipids. The resulting organic phase was carefully separated and dried after centrifugation (4000 rpm) for 10 min. The dried mixture was re-dissolved in 10 ml chloroform for purification process.

### Quantitative Analysis of Synthesized Phosphatidyl Saccharides

The molar yield of phosphatidyl saccharides was determined by HPLC analysis. The HPLC was equipped with a silica gel column (5 µm, 4.6×250 mm, Thermo Fisher Scientific Inc.) and a Sedex (Alfortville, France) model 75 ELSD; the pressure of nebulizer gas (air) was maintained at 3.2 bar and the drift tube temperature was set at 40 °C. Three kinds of eluents namely chloroform (A), methanol (B), and 1% (V/V) triethylamine buffer (titrated at pH 3 with formic acid) (C) were used. The chromatographic separation was carried out using a linear gradient: 0-5 min A/B/C (87.5/12/0.5, v/v), 5-40 min A/B/C (87.5/12/0.5, v/v) to A/B/C (28/60/12, v/v), 40-45 min back to A/B/C (87.5/12/0.5, v/v), and 45-55 min re-equilibration. The flow rate of the eluent was 0.5 ml/min. The retention time of Ptd-Glu, Ptd-Suc and Ptd-Raff were 27.1 min, 28.2 min and 29.7 min, respectively. Phosphatidyl saccharides were quantified with standard curve of purified phosphatidyl saccharides, whose structure and purity have been confirmed by LC/MS and NMR analysis.

### Purification of Phosphatidyl Saccharides from Reaction Mixture

The synthesized phosphatidyl saccharides were separated from extracted polar lipids according to a reported TLC method with slight modification [20]. Around 0.5 ml of the lipid extraction from reaction mixture was separated on thin layer chromatography plates (TLC silica gel 60, 10×20, Merck, Germany) using developing solvent of chloroform/methanol/acetate acid/water (50:25:6:2). The R_f_ value for Ptd-Glu, Ptd-Suc and Ptd-Raff in TLC were 0.48, 0.26 and 0.09 respectively. The phosphatidyl saccharide band was scraped off and extracted by chloroform/methanol (1:1). The extract was dried after centrifugation (4000 rpm) for 20 min. The purity of extracted phosphatidyl saccharide was reconfirmed by TLC and HPLC with single plot and single peak, respectively. The structure of phosphatidyl saccharides was further identified by LC-ESI-FTMS and NMR.

### Structure Conformation of Phosphatidyl Saccharides

The structures of the synthesized phosphatidyl saccharides (Ptd-Glucose, Ptd-Sucrose, and Ptd-Raffinose) were identified by NMR and LC/FTMS.

**NMR** investigations on Ptd-Glu was carried out at 14.1 T on a Bruker Avance III spectrometer in an inverse TXI (^1^H,^31^P,^13^C) 1.7 mm probe with a z-gradient at 300 K. In order to verify the phosphor-ether bond a ^31^P-^1^H HMBC pulse sequence was employed. The sequence was based on the standard Bruker parameter set HMBCGPND and optimized for 4 Hz couplings. The 64 indirect increments each with four scans were acquired and zero filled to 8192 times 128. Gradients in the pulse sequence were set in the ratio 20:30:10.16 in order to select for ^31^P-^1^H couplings. The ^31^P dimension was referenced externally to phosphatidylcholine in CDCl_3_:MeOD (2:1) at 0.8 ppm and the proton dimension was references to TMS at 0 ppm in CDCl_3_:MeOD (2:1). For assignment purposes HSQC, DQF-COSY, TOCSY and J-resolved spectra was also recorded all using standard parameters. For the Ptd-Suc and Ptd-Raff samples, cryogenic probes and RT probes where used. The following experiments, ^13^C-^1^H HSQC, ^13^C-^1^H HSQCTOCSY and ^1^H-^31^P TOCSY was recorded in order to determine the phosphorus linkage all based on standard bruker pulse programs. The ^1^H-^31^P TOCSY utilized a 60 µs spinlock for 80 ms.

**LC/FTMS** includes chromatographic separation (Agilent 1100 System consisting of G1376A (Cap pump), G1377A (µWell-Plate Sampler), G1316A (Column Compartment) and FTMS detection (LTQ Orbitrap MS, Thermo Scientific). The chromatographic separation was obtained by normal-phase conditions using a Kromasil Sil column, 150 mm x 0.5 mm id., 3.5 µm. The mobile phases were: Solvent A comprised of chloroform/MeOH/NH _4_OH (25%) (800/200/5 vol.) and solvent B comprised of chloroform/MeOH/H _2_O/NH _4_OH/ (25%) (800/200/55/5 vol.). The flow rate was 16 µl/min. The gradient is 100% A (0-5 min), 100% A-100% B (5-10 min), 100% B (10-25 min), 100% B-100% A (25-32 min), and 100% A (32-40 min) for equilibration. The total cycle time was 40 min. The MS condition was negative electrospray mode and two scan events pr cycle: 1) Full scan FTMS [200-1400] with 15 k resolution, 2) data dependant FTMS^2^ with 7.5 k resolution.

### Preparation of Nanoliposome Suspension of Phospholipids by Thin Film Hydration Technique

Solid phospholipids (1 mg) were dissolved in 1 ml chloroform: methanol (1:1). For CLSM analysis, 0.1 ml of a 0.1 mM dichloromethane solution of DIL (1,1’-Dioctadecyl-3,3,3’,3’- tetramethylindocarbocyanine perchlorate) was added to the formulation. The solvent was evaporated by a stream of dry nitrogen until a dry lipid film was observed and followed by overnight vacuum drying to remove all solvent. Following overnight vacuum drying, the liposomes were suspended in Milli-Q water by vigorous vortexing for 30 mins and allowed to hydrate at room temperature for 3h.

### Characterization of Physical Properties

Physical property of phosphatidyl saccharides and PC nanoliposomes were evaluated by critical micelle concentration (CMC), dynamic light scattering (DLS), confocal laser scan microscope (CLSM), and atom force microscope (AFM).

**Critical Micelle Concentration (CMC**) measurement was done using the pyrene assay [21]. Fluorescence spectrometer (PerkinElmer LS55 luminescence spectrometer) was used in this study. Aggregate formation was examined over a wide range of phospholipid concentration (1×10^-7^-1 mg/ml) by using pyrene in methanol as a fluorescent probe (final concentration 1×10^-6^ M). Brieﬂy, the ratio between the emission intensity at two wavelengths (I_1_=372.5 and I_3_=383 nm) upon excitation at 335 nm was determined. I_1_/I_3_ is around 1.0 in the absence of surfactant but increases to a plateau value of 1.42-1.53 in the presence of micelles, depending on the speciﬁc surfactant headgroups. The CMC was taken as the intersection of regression lines calculated from the linear portions of the graph.

**Dynamic Light Scattering (DLS**) was performed at 25 °C. The diameter and zeta potential of liposome were characterized by dynamic light scattering (DLS) using a Malvern Zetasizer Nano (Malvern Instruments Ltd., Worcestershire, UK). All measurements were performed at an angle of 173°. The size distributions and zeta potentials were analyzed by using the Malvern Dispersion Software (V5.10, www.zetasizer.com). Since size changes observation is easier with dispersion with low polydispersity index (PDI), the phospholipid suspensions from shelf life evaluation were passed through a 0.45 µm PTFE syringe filter (Whatman).

**Confocal Laser Scan Microscopy (CLSM**)** Analysis** A Zeiss 510 Meta CLSM microscope with a 100x oil immersion 1,3 NA objective was used. The DIL dye was excited with the 543 line of the He-Ne laser and the emission was captured with the 560-620 nm BP filter. The liposome was fixed on 2% (W/V) agarose gel. To observe dehydrating procedure, the fixed liposome was dried on cover slip in ambient condition overnight. To observe rehydrating procedure, the dehydrated sample was rehydrated in Milli-Q water for 1 h.

**Atom Force Microscopy (AFM**)** Analysis** A commercial AFM MultiMode V (Bruker, Santa Barbra, USA) was used. For sample preparation, 5 µL of PC, Ptd-Glu, Ptd-Suc or Ptd-Raff with the concentration of 2 mg/ml were deposited on the freshly cleaned mica for 10 min, after that the residue solution were removed. All the samples were washed 3 times with Milli-Q water and dried in ambient condition before the measurement. The standard AFM images were recorded using tapping mode under ambient conditions. Commercial silicon tips from Bruker Company (Bruker, Santa Barbra, USA) with a nominal spring constant of 40 N/m and resonant frequency of 300 kHz were used in all the experiments and a normal tip radius of 15 nm were chosen to image the samples. All the AFM images were obtained in the tapping mode at a scan frequency of 1 Hz with optimized feedback parameters. The resolution of all the original AFM images was 512 x 512 pixels per image. The images were flattened and analyzed by using the commercial software Scanning Probe Image Processor (SPIPTM, Image Metrology Aps, version 5.13, Lyngby, Denmark).

## Results and Discussion

### Synthesis and Structural Mapping of Phosphatidyl Saccharides

**Figure 1:**
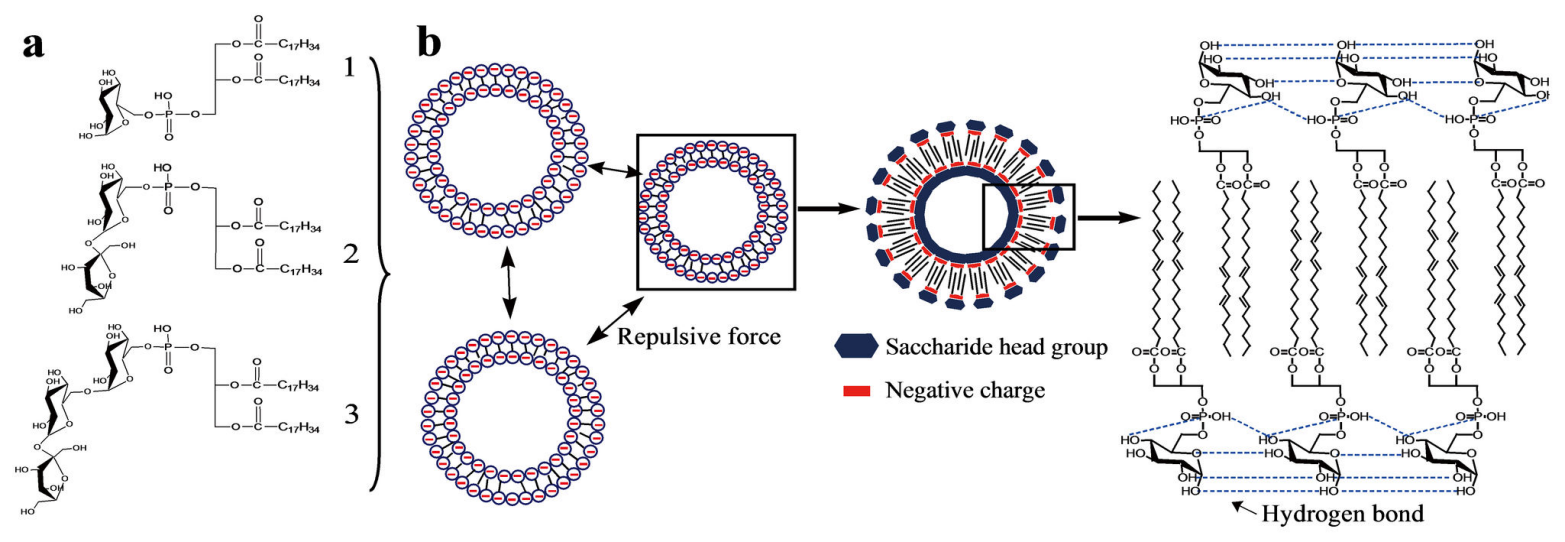
Phosphatidyl saccharides and the mechanism concerning stabilization of phosphatidyl saccharides nanoliposomes during storage and dehydration/rehydration. (a) Structure of enzymatic synthesized phosphatidyl saccharides (1:Ptd-Glu; 2:Ptd-Suc; 3:Ptd-Raff). (b) Both the electrostatic repulsive force caused by negative surface charge and the enhanced hydrogen bond between saccharide head groups contributed to the improved stability of phosphatidyl saccharides nanoliposomes.

One of the major challenges in synthesizing phosphatidyl saccharides is the selectivity because of presence of multiple hydroxyl groups in sugar ring. Taking advantage of high selectivity of biocatalysis, we employed PLD to catalyze the transphosphatidylation of PC with saccharides (glucose, sucrose and raffinose) in a binary ethyl acetate/water system. This binary solvent system is able to dissolve both phophostidylcholine and sugar, respectively [22]. 95 mol% of Ptd-Glu can be obtained at optimum condition. Substrates with bigger molecular size have higher steric hindrance and thus difficult to access the active site of enzyme. Therefore lower molar yields were obtained for Ptd-Suc (67 mol%) and Ptd-Raff (23 mol%). Figure 1 (a) shows the structures of the synthesized anionic phosphatidyl saccharides.

**Figure 2:**
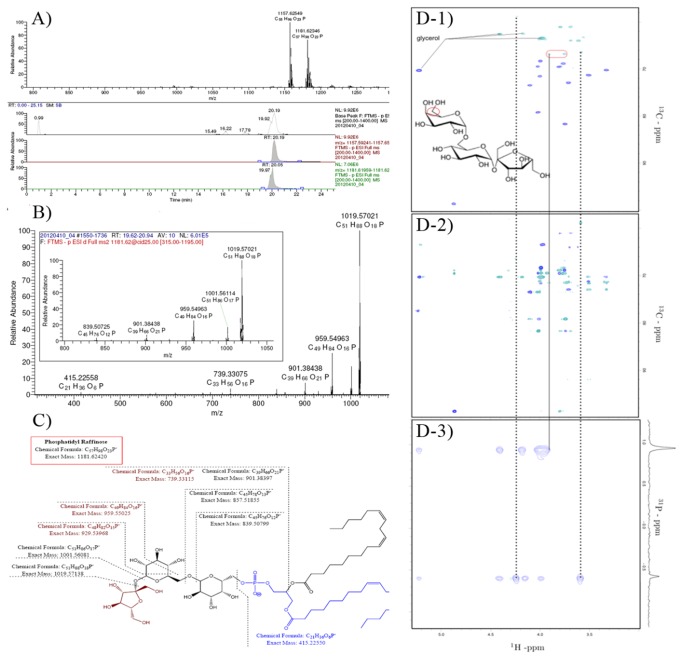
Structural elucidation of Ptd-Raff. A) Base-peak and extracted ion chromatograms and full FTMS spectrum of Ptd-Raff. The two major isomers are m/z 1181 [18:2, 18:2] and m/z 1157 [18:2, 16: 0]. B) MS^2^ spectra of the two major isomers. The primary fragments are explained in C). C) Chemical structure of Ptd-Raff. D-1) multiplicity edited ^13^C-^1^H HSQC, D-2) multiplicity edited ^13^C-^1^H HSQC-TOCSY and D-3) ^1^H-^31^P TOCSY with ^31^P detection spectra of Ptd-Raff. Dotted lines shows correlation confirming small amount of phosphatidylcholine in the Ptd-raffinose sample (Ratio estimated by quant. ^31^P NMR to 80:20 Ptd-Raff/PC. The solid line in shows the major phosphor species in Ptd-raffinose conjugate on the galactose unit of raffinose.

The chemical structures of the purified phosphatidyl saccharides were confirmed by LC/FTMS and NMR (Figure 2 for Ptd-Raff; Figures S1 and S2 in Supplementary Materials for Ptd-Glu and Ptd-Suc, respectively). LC/FTMS analysis showed all samples were of high purity and the molecular mass for all purified phosphatidyl saccharides were as predicted. Two abundant isomers can be found for all phosphatidyl saccharides which can be attributed to the differences in the fatty acid chains of the substrate PC ([18:2, 18:2], [16: 0, 18:2]). For example, Ptd-Raff presented m/z 1181.62 [18:2, 18:2] and m/z 1157.62 [18:2, 16: 0] (Figure 2 A). MS^2^ spectra further verified the structure of all the phosphatidyl saccharides as the typical fragments include loss of the glycan unit, fatty acids and C_2_H_2_O_2_ by glucan-ring cleavage. For example, fragments of 1019.57 or 1001.56 (fatty acids-glycan unit-C _2_H_2_O_2_) and 959.55 (fatty acids-glycan unit) were observed in Ptd-Raff (Figure 2 B-C). NMR Spectroscopy was used to assign the molecular moieties of the phosphatidyl saccharides. ^31^P-^1^H HMBC, ^1^H-^31^P TOCSY and ^13^C-^1^H HSQC NMR pulse sequences verified the connectivities of the carbohydrate functionality to the Ptd-backbone (Figure 2 D). For Ptd-Glu and Ptd-Raff, the phosphate ester of the Ptd-backbone binds to glucose-C6 and galactose-C6, respectively. A similar connectivity is dominant for Ptd-Suc, phosphorus was more selectively linked with glucose-C6 of sucrose, but less intense correlations (~20%), are also observed to fructose-C2 of sucrose (Figure S2D).

### Physical Characteristics and Storage Stability of Phosphatidylsaccharide-based Nanoliposomes

Commercial PC and the purified synthetic phosphatidyl saccharides were used to prepare nanoliposomes. The physical characteristics of nanoliposomes were evaluated (Table 1). Phosphatidyl saccharide-based nanoliposomes were smaller in size than the PC based ones. Saccharides have higher optimal headgroup area as compared to choline; therefore, phosphatidyl saccharides have smaller packing parameter which resulted in higher membrane curvature and smaller vesicle size [23]. In terms of surface charge, all the phosphatidyl saccharides-based nanoliposomes had negative surface charge (negative zeta potential) due to the negatively-charged phosphate group. Meanwhile, PC nanoliposomes had neutral charge due to the zwitterionic structure of PC. The anionic phosphatidyl saccharide nanoliposomes provided repulsive forces which prevent fusion and aggregation. A possible mechanism has been proposed as indicated in Figure 1(b).

**Table 1:** Critical micelle concentration (CMC), liposome average size and zeta potential of PC, Ptd-Glu, Ptd-Suc and Ptd-Raff.

	CMC (nM)	Vesicle Size (nm)	Zeta (mV)
PC	0.12	908.70 ± 1.21	-9.50 ± 0.65
Ptd-Glu	0.16	290.57 ± 2.56	-92.10 ± 3.50
Ptd-Suc	0.18	126.87 ± 4.74	-91.20 ± 2.56
Ptd-Raff	0.20	141.63 ± 1.66	-91.43 ± 0.51

Both phosphatidyl saccharides and PC nanoliposomes had similar critical micelle concentration (CMC). As CMC is strongly dependent on the alkyl chain length (hydrophobicity), the property change in headgroup despite insignificant still influences the CMC [24]. Increasing amount of sugar ring from glucose to raffinose (hydrophilic moiety) resulted in 1.1 to 1.3 times increments in CMC per glycan unit (Table 1).

### Storage Stability of Phophatidyl Saccharide-based Nanoliposomes

**Figure 3:**
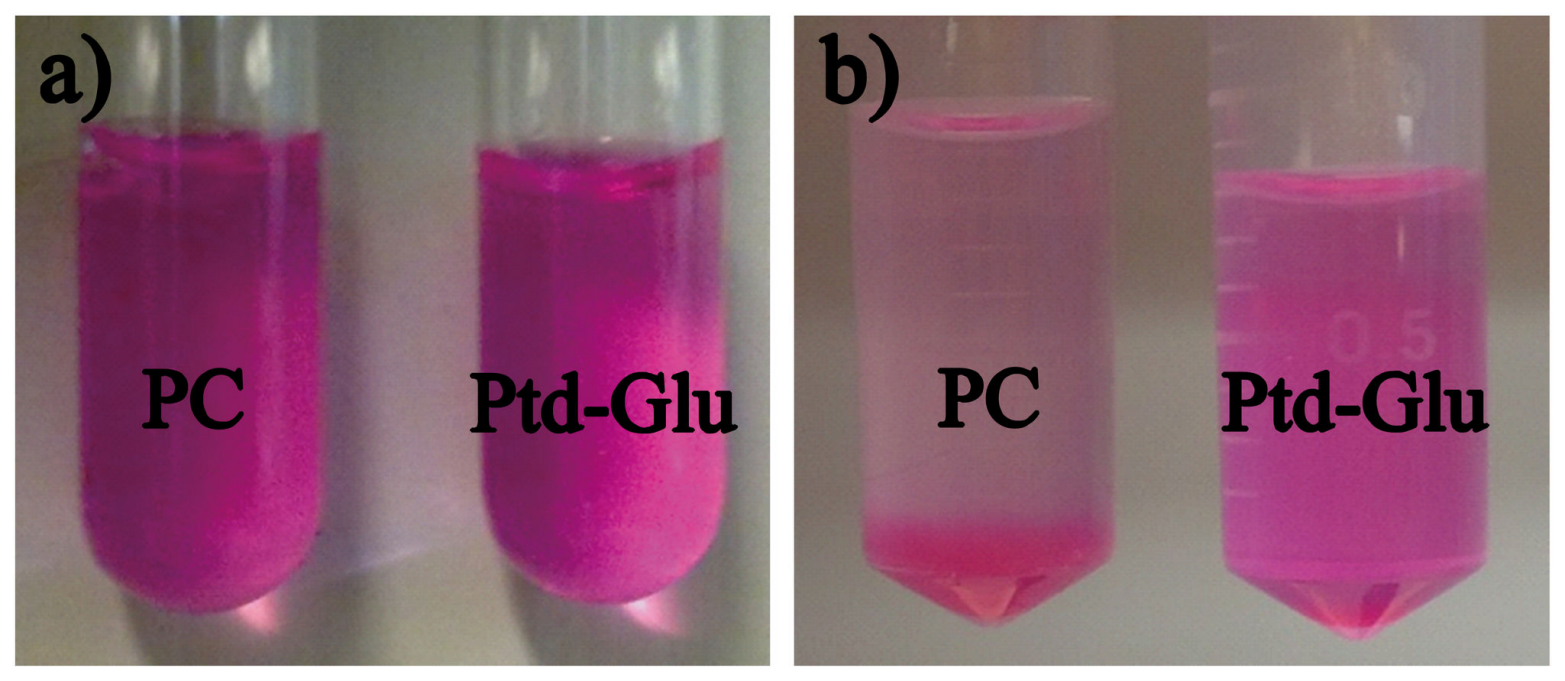
Physical stability of PC and phsphatidyl saccharide nanoliposomes following prolonged storage: a) freshly prepared nanoliposomes, b) nanoliposome after storage for 14 days at room temperature.

**Figure 4:**
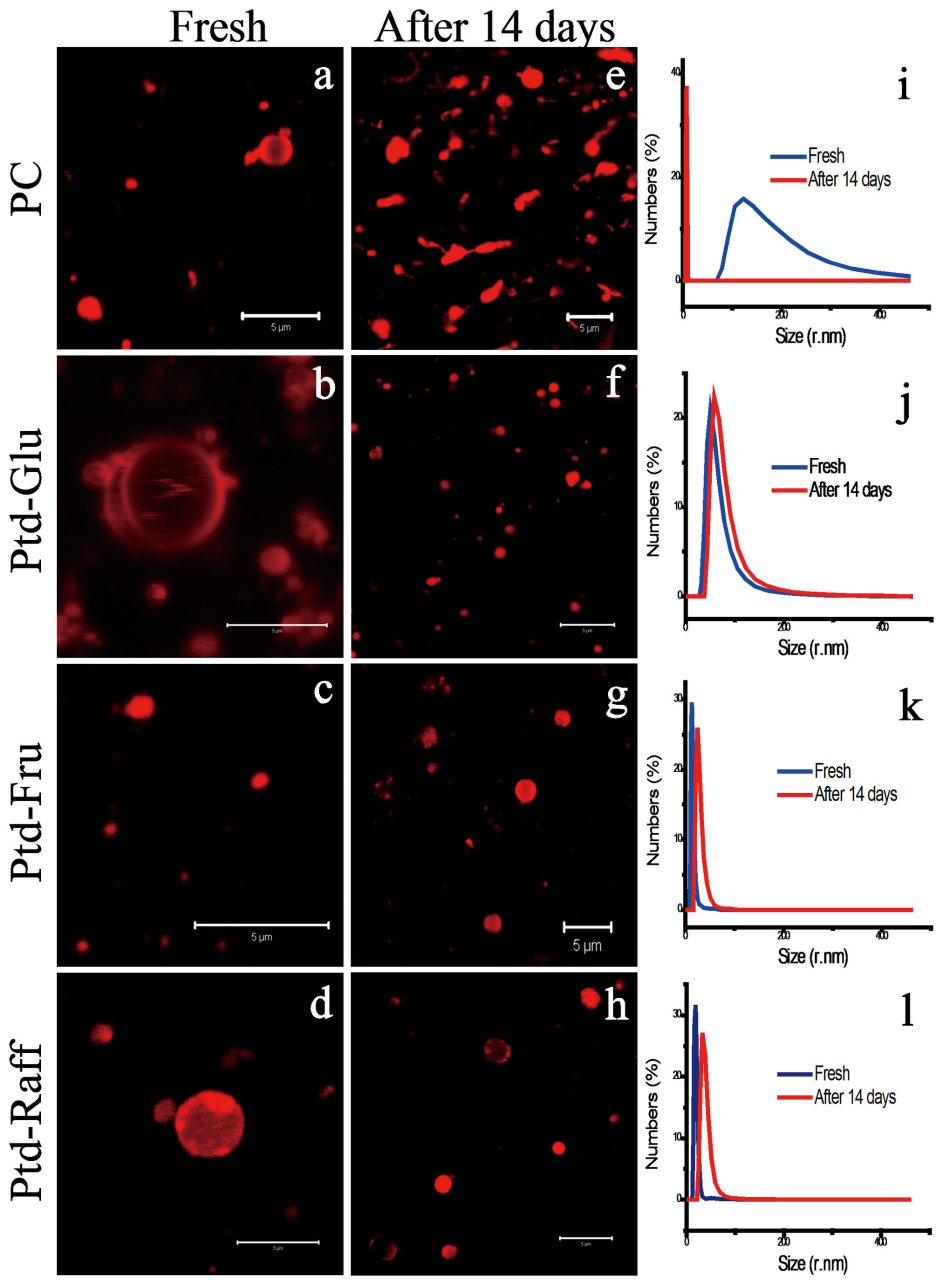
Characterization of nanoliposomes of PC, Ptd-Glu, Ptd-Suc and Ptd-Raff following prolonged storage by CLSM and DLS. (a-d) Morphology of freshly prepared nanoliposomes (size bar: 5 µm). (e-h) Morphology of nanoliposomes following storage for 14 days at room temperature (size bar: 5 µm). (i-l) Size dispersion of freshly prepared nanoliposomes and nanoliposomes stored for 14 days.

The nanoliposomes of phosphatidyl saccharides and PC loaded with hydrophobic fluorochrome marker 1,1-Dioctadecyl-3,3,3’,3’-tetramethyl indocarvicyanine perchlorate (DIL) were prepared and stored for 14 days at room temperature. For phosphatidyl saccharides nanoliposomes, no apparent precipitation and aggregation is observed after 14 days’ storage in comparison with the freshly prepared samples (Figure 3). Meanwhile, PC nanoliposomes aggregated and fused into bigger droplets which eventually precipitated (Figure 3). Materials with small particle size distribution of less than 10 nm can be observed in the aqueous suspension of PC which may represents the collapsed membrane pieces or micelle. However, only a little bit difference of the size distribution of Ptd-saccharide based nanoliposomes between the fresh sample and the sample after storing 14 days was observed (Figure 4). The stability of phosphatidyl saccharides nanoliposomes against aggregation and fusion during storage was further verified using Confocal Light Scattering Microscopy analysis (CLSM). The nanoliposomes loaded with hydrophobic fluorochrome marker DIL were fixed on agarose gel. After storage for 14 days, the DIL-labelled phosphatidyl saccharides nanoliposomes remained intact which can be shown by the unilamellar red-stained spherical morphology (Figure 4). In contrast, DIL-labelled PC nanoliposomes were characterized as red-stained elongated and irregular morphology which indicate aggregation and fusion of the nanoliposomes. One of the possible reasons for higher stability of phosphatidyl saccharide nanoliposomes against aggregation and fusion during storage is the OH groups of saccharide moiety. The OH-groups are capable of forming abundant hydrogen bond in the polar region of the membrane resulting in enhanced membrane mechanical stability. Surface charge also plays an important role. Liposomes formed by neutral sugar esters were found to aggregate during storage due to the low head group repulsive forces [25]. Thus, the anionic phosphatidyl saccharides nanoliposomes provided repulsive forces between the headgroups to prevent aggregation and fusion of nanoliposomes (Figure 1b).

### Stability of Phosphatidylsaccharide-based Nanoliposomes against Dehydration/Rehydration

**Figure 5:**
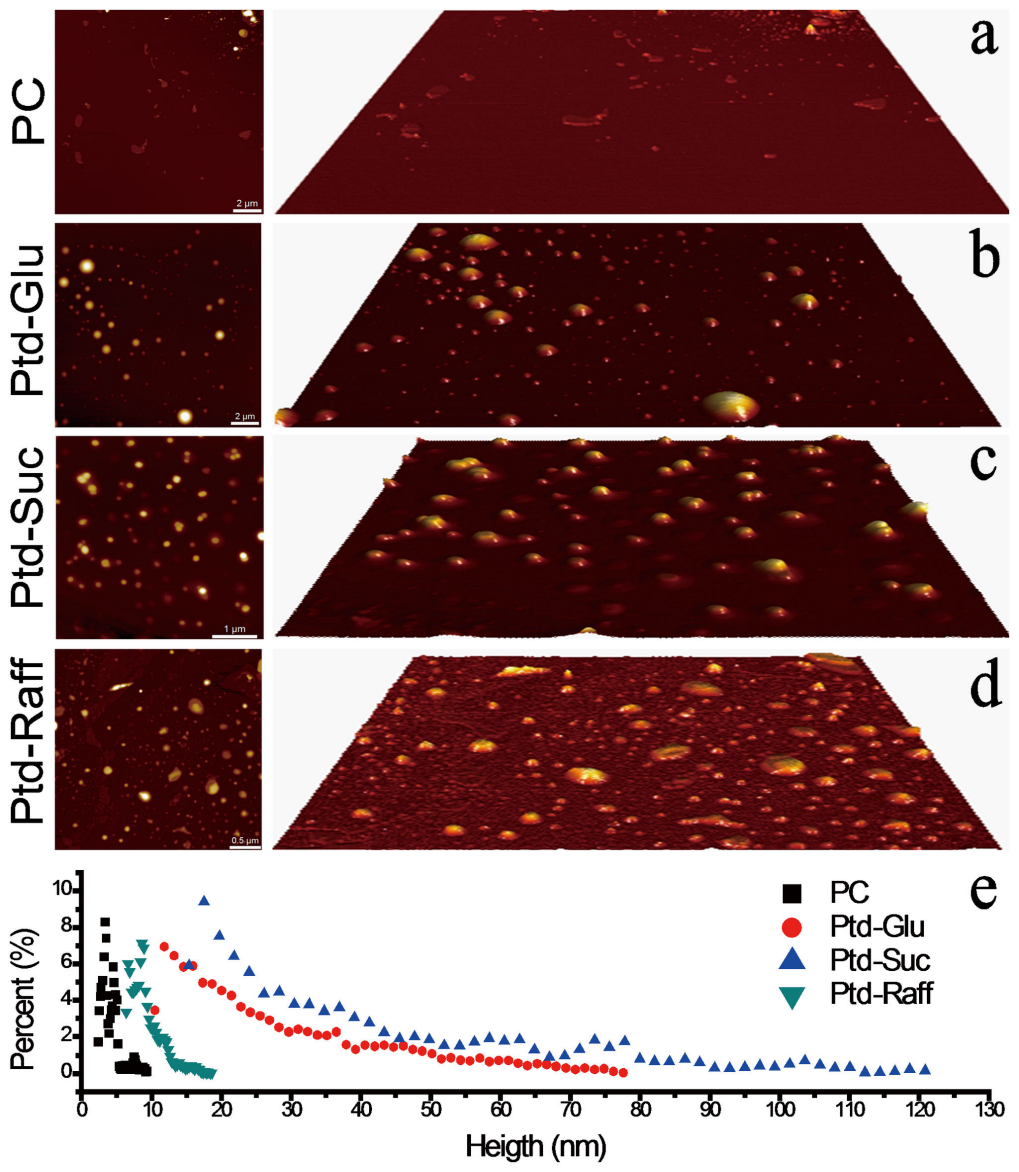
AFM topographic images of the nanoliposomes of PC (a), Ptd-Glu (b), Ptd-Suc (c) and Ptd-Raff (d) on mica surface following dehydration procedure. The height distribution of these structures were depicted in (e).

**Figure 6:**
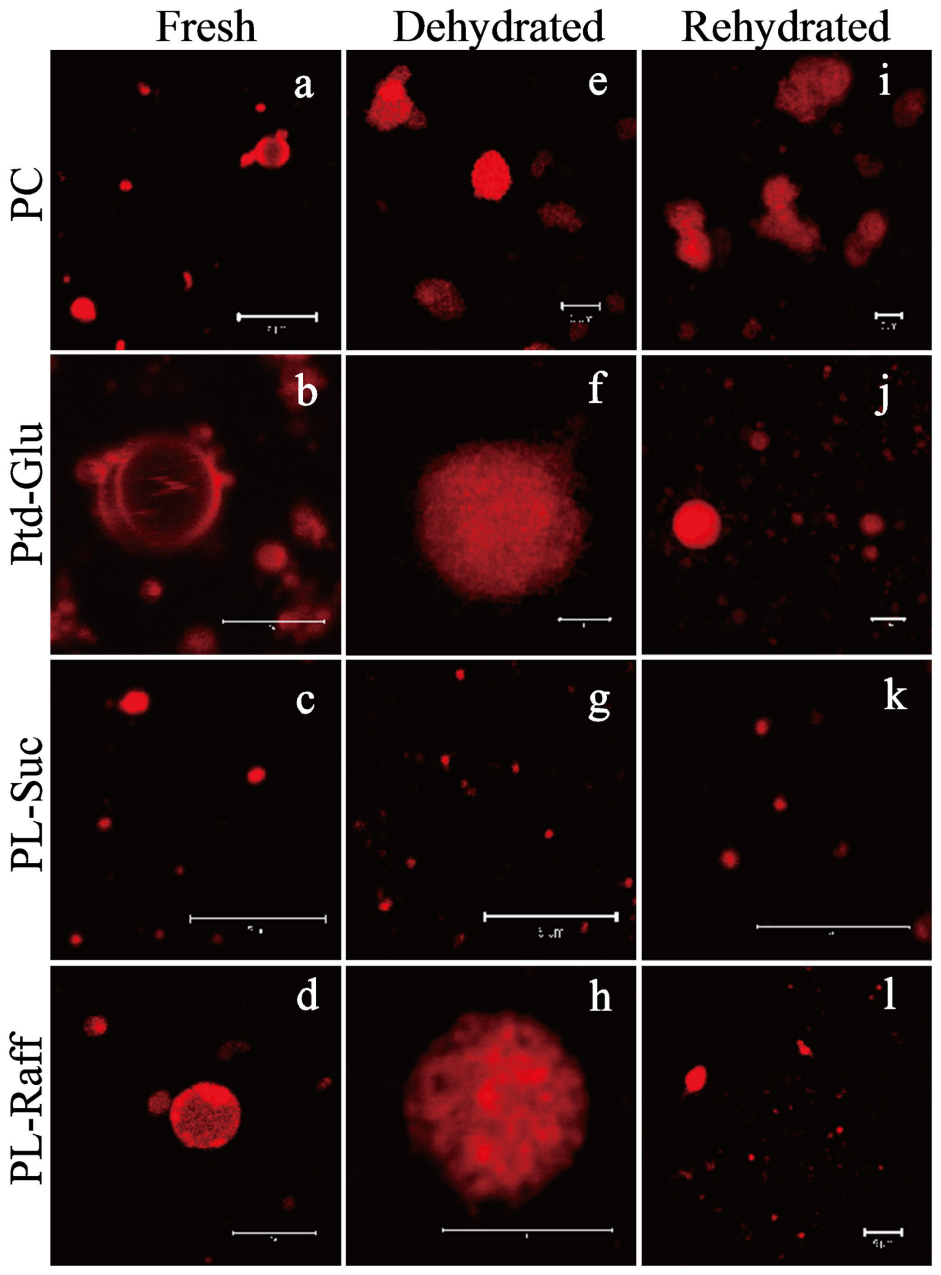
CLSM images of nanoliposomes following dehydration and rehydration procedure (size bar: 5 µm). (a-d) Morphology of freshly prepared nanoliposomes (e-h) Morphology of nanoliposomes following dehydration procedure (i-l) Morphology of nanoliposomes following rehydration procedure.

Phosphatidyl saccharide nanoliposomes also demonstrated improved stability against dehydration and rehydration procedure. Following dehydration, most of the phosphatidyl saccharide nanoliposomes remained intact on the hydrophilic mica surface. Both Ptd-Glu and Ptd-Suc nanoliposomes had a height distribution within 18-80 nm and 30-130 nm, respectively. Ptd-Raff nanoliposomes had a height distribution of less than 20 nm. In comparison, nearly all of the PC nanoliposomes collapsed to form bilayer structure on the mica surface (height<10 nm) (Figure 5). Similar findings can be observed using the CLSM (Figure 6). The DIL-labelled phosphatidyl saccharide nanoliposomes remained intact with apparent red-stained spherical morphology following dehydration. Upon rehydration, the DIL-labelled phosphatidyl saccharide nanoliposomes can be recovered. Nevertheless, Ptd-Raff had reduced vesicle size upon rehydration procedure which is in agreement with the aforementioned AFM findings. Stark et al. [26] found stabilizing effects of protectants is strongly dependent on its water solubility. Protectants with higher water solubility have higher OH bonding capability. In present case, sucrose (2000g/L) has the highest water solubility followed by glucose (910g/L) and raffinose (143g/L). Thus, Ptd-Suc has the highest stabilizing effect followed by Ptd-Glu and Ptd-Raff. In comparison, the DIL-labelled PC nanoliposomes aggregated and fused (red-stained elongated and irregular morphology) upon dehydration. Rehydration procedure was not able to recover the liposomal structures of the DIL-labelled PC nanoliposomes.

## Conclusions

In summary, a series of anionic phosphatidyl saccharides were successfully synthesized through enzymatic transphosphatidylation. Phosphatidyl saccharide nanoliposomes were smaller in size and had higher negative surface charge as compared to PC nanoliposomes. Presence of abundant OH groups in phosphatidyl saccharides renders them stabilizing effects. Anionic phosphatidyl saccharides nanoliposomes demonstrated enhanced physical stability against fusion and aggregation following storage, dehydration and rehydration procedures. The findings from this study can be used to overcome the physical instability of liposomes. And the synthetic phosphatidyl saccharides may provide an alternative anionic liposome for non-cytotoxic and serum stable delivery system [27]. In addition, sugar-coated nanoparticles as delivery system are becoming an area of interest since their reported cell internalization ability [28] and specific molecular recognition properties [29]. Thus, phosphatidyl saccharide nanoliposomes may have similar effects and can be used for targeted drug or functional ingredients delivery. More study on functional molecular encapsulation and cell internalization of the phosphatidyl saccharide liposomes will be further carried out.

## Supporting Information

Figure S1 Structural elucidation of Ptd-Glu. (**A**) Base-peak and extracted ion chromatograms and full FTMS spectrum of Ptd-Glu. (**B**) MS^2^ spectra of the two major isomers. The primary fragments are the loss of the glycan-unit, loss of C_2_H_4_O_2_ from the glycan ring and loss of glycan and one fatty acid. (**C**) Structure of Ptd-Glu (D-1). ^13^C-^1^H HSQC spectrum of Ptd-Glu, showing chemical shifts of the α- and β-conformer of the glucose unit (D-2). ^31^P-^1^H HMBC spectrum of Ptd-Glu, showing correlations between phosphorous and the$H_{\alpha }^{6}$, $H_{\beta }^{6}$ and the$H_{\alpha }^{glycerol}$ protons.
(TIF)

Figure S2 Structural elucidation of Ptd-Suc. (**A**) Base-peak and extracted ion chromatograms and full FTMS spectrum of Ptd-Suc. (**B**) MS^2^ spectra of the two major isomers. The primary fragments are the loss of the glycan-unit, loss of C_2_H_4_O_2_ from the glycan ring and loss of glycan and one fatty acid (**C**) Structure of Ptd-Suc (**D-1**). multiplicity edited ^13^C-^1^H HSQC, (**D-2**) multiplicity edited ^13^C-^1^H HSQC-TOCSY and (**D-3**) ^1^H-^31^P TOCSY with ^31^P detection spectra of Ptd-Suc. The solid line shows the major phosphor species in Ptd-Suc conjugate on 6-OH (C6) of glucose unit and only small amount of phosphor species conjugate on 2-OH (C2) of fructose unit. The phosphor peaks are in 72:20:8 ratio measured by quantitative ^31^P NMR.
(TIF)
