# Supplementary figures and images for: Facile Synthesis of Phosphatidyl Saccharides for Preparation of Anionic Nanoliposomes with Enhanced Stability
Source: PLoS One. 2013 Sep 12;8(9):e73891. doi: 10.1371/journal.pone.0073891 (PMC3771975; doi:10.1371/journal.pone.0073891)

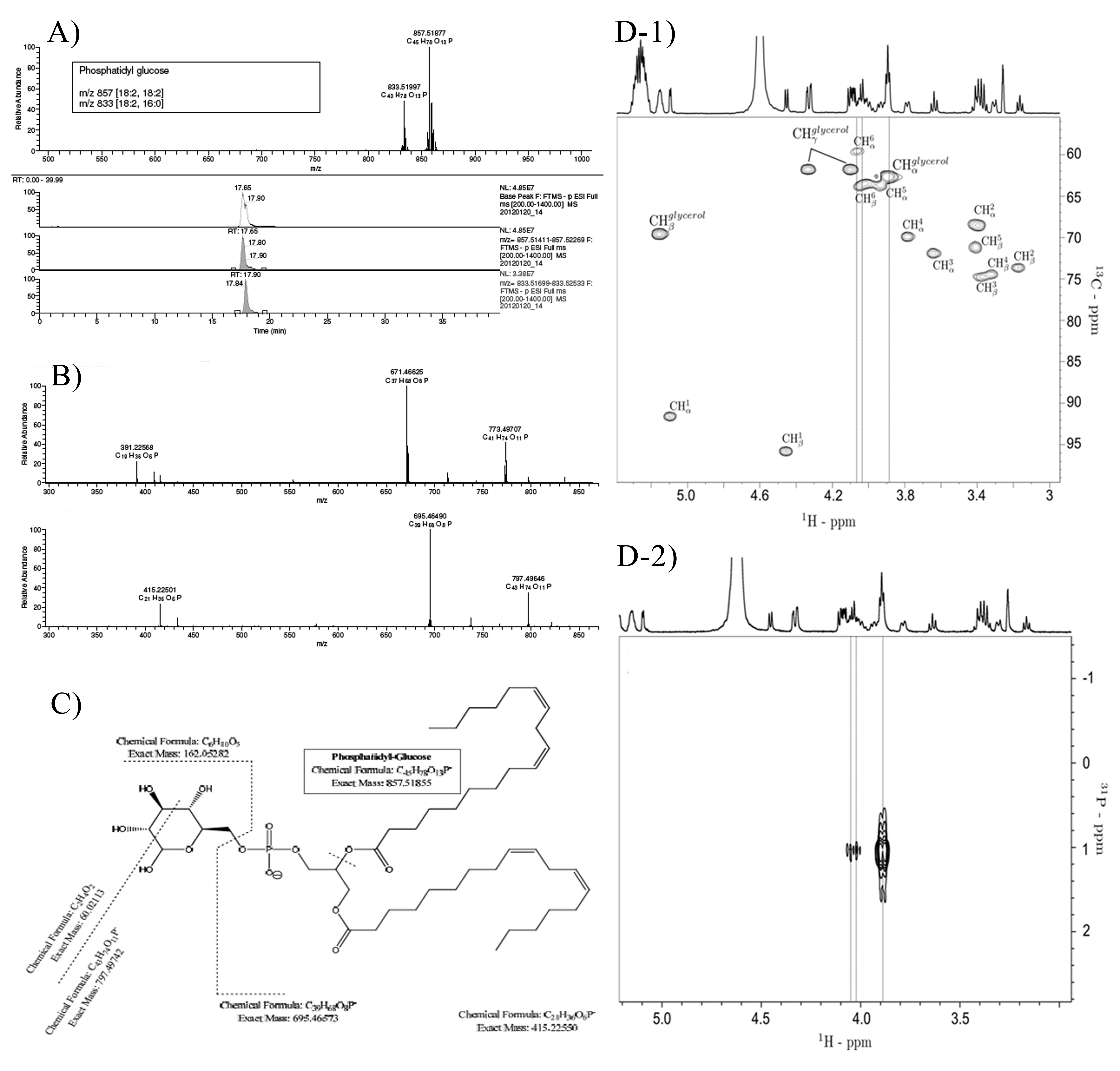

Supplement: Figure S1 — Structural elucidation of Ptd-Glu. (A) Base-peak and extracted ion chromatograms and full FTMS spectrum of Ptd-Glu. (B) MS2 spectra of the two major isomers. The primary fragments are the loss of the glycan-unit, loss of C2H4O2 from the glycan ring and loss of glycan and one fatty acid. (C) Structure of Ptd-Glu (D-1). 13C-1H HSQC spectrum of Ptd-Glu, showing chemical shifts of the α- and β-conformer of the glucose unit (D-2). 31P-1H HMBC spectrum of Ptd-Glu, showing correlations between phosphorous and theHα6, Hβ6 and theHαglycerol protons. (TIF) [file pone.0073891.s001.tif]

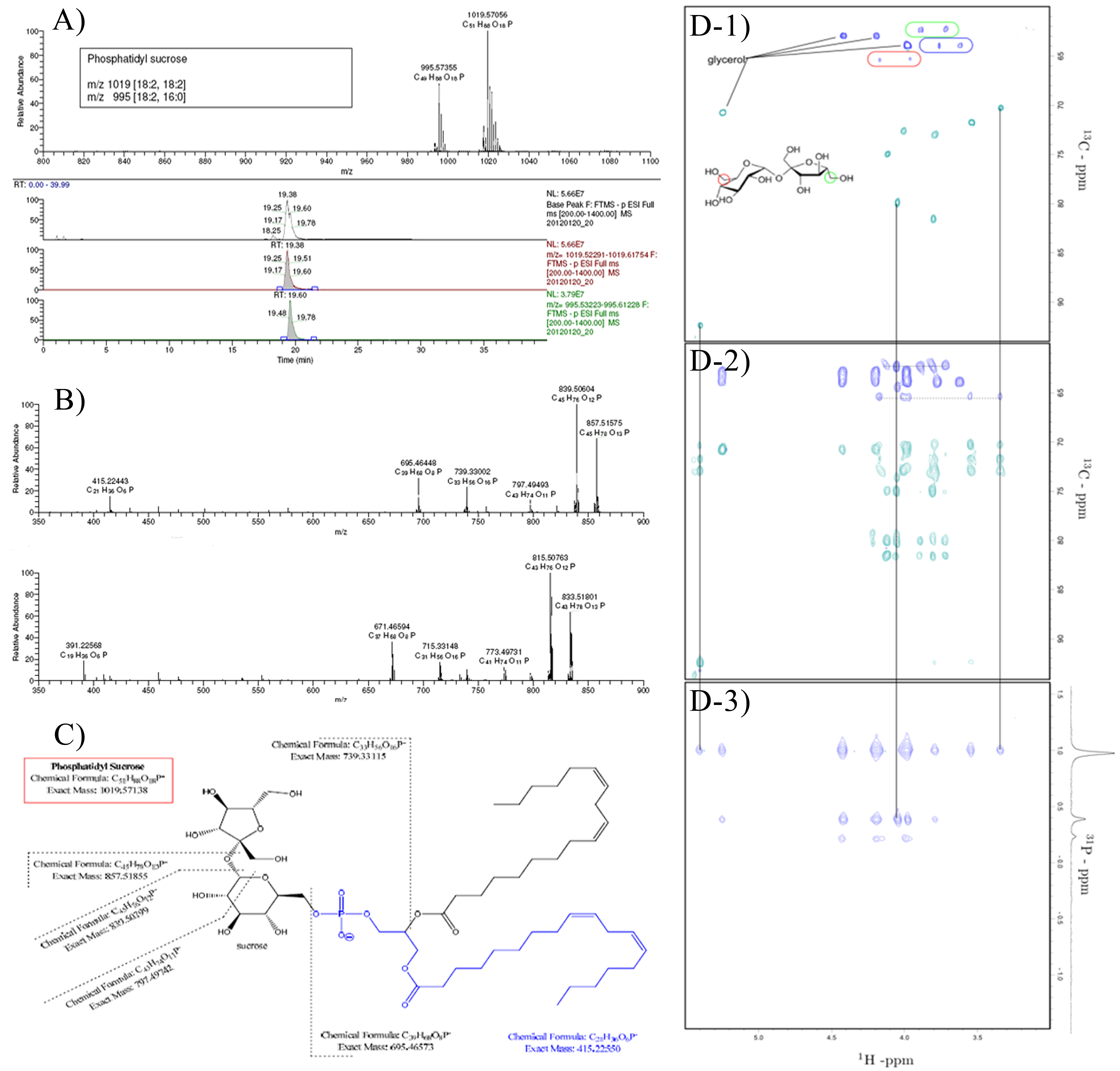

Supplement: Figure S2 — Structural elucidation of Ptd-Suc. (A) Base-peak and extracted ion chromatograms and full FTMS spectrum of Ptd-Suc. (B) MS2 spectra of the two major isomers. The primary fragments are the loss of the glycan-unit, loss of C2H4O2 from the glycan ring and loss of glycan and one fatty acid (C) Structure of Ptd-Suc (D-1). multiplicity edited 13C-1H HSQC, (D-2) multiplicity edited 13C-1H HSQC-TOCSY and (D-3) 1H-31P TOCSY with 31P detection spectra of Ptd-Suc. The solid line shows the major phosphor species in Ptd-Suc conjugate on 6-OH (C6) of glucose unit and only small amount of phosphor species conjugate on 2-OH (C2) of fructose unit. The phosphor peaks are in 72:20:8 ratio measured by quantitative 31P NMR. (TIF) [file pone.0073891.s002.tif]
